# Determining Ribavirin’s mechanism of action against Lassa virus infection

**DOI:** 10.1038/s41598-017-10198-0

**Published:** 2017-09-15

**Authors:** Paola Carrillo-Bustamante, Thi Huyen Tram Nguyen, Lisa Oestereich, Stephan Günther, Jeremie Guedj, Frederik Graw

**Affiliations:** 1 Center for Modeling and Simulation in the Biosciences (BIOMS), BioQuant-Center, Heidelberg University, Heidelberg, Germany; 2 INSERM, IAME, UMR, 1137 Paris, France; 3 Université Paris Diderot, IAME, UMR, 1137 Sorbonne Paris Cité, France; 4 Bernhard-Nocht-Institute for Tropical Medicine, Hamburg, Germany; 5 German Center for Infection Research (DZIF), Partner Site Hamburg, Germany

## Abstract

Ribavirin is a broad spectrum antiviral which inhibits Lassa virus (LASV) replication *in vitro* but exhibits a minor effect on viremia *in vivo*. However, ribavirin significantly improves the disease outcome when administered in combination with sub-optimal doses of favipiravir, a strong antiviral drug. The mechanisms explaining these conflicting findings have not been determined, so far. Here, we used an interdisciplinary approach combining mathematical models and experimental data in LASV-infected mice that were treated with ribavirin alone or in combination with the drug favipiravir to explore different putative mechanisms of action for ribavirin. We test four different hypotheses that have been previously suggested for ribavirin’s mode of action: (i) acting as a mutagen, thereby limiting the infectivity of new virions; (ii) reducing viremia by impairing viral production; (iii) modulating cell damage, i.e., by reducing inflammation, and (iv) enhancing antiviral immunity. Our analysis indicates that enhancement of antiviral immunity, as well as effects on viral production or transmission are unlikely to be ribavirin’s main mechanism mediating its antiviral effectiveness against LASV infection. Instead, the modeled viral kinetics suggest that the main mode of action of ribavirin is to protect infected cells from dying, possibly reducing the inflammatory response.

## Introduction

Lassa fever (LF) is a severe and often fatal hemorrhagic disease caused by Lassa virus (LASV), a member of the *Arenaviridae* virus family. LASV is endemic in West Africa, causing over 200.000 infections annually, resulting in several thousands of deaths^1–3^. Although case fatality rates among hospitalized LF patients can exceed 50%, numerous infections are mild or even asymptomatic^2^. There is currently no vaccine available against LASV in humans, and the sole treatment relying on the drug ribavirin is only effective if administered early in infection, i.e., within the first 6 days after the onset of clinical symptoms^4^.

Ribavirin is a guanosine analogue displaying broad antiviral activity against several RNA and DNA viruses^5,6^. It efficiently suppresses the replication of LASV *in vitro* ^7^, yet the drug’s efficacy in reducing viremia *in vivo* is moderate, causing instead large declines in the levels of aminotransferases (ALT, and AST), effectively reducing cell damage^7^. However, ribavirin significantly improves clinical outcome, i.e., survival, during LASV infection in mice when given as a combination therapy with favipiravir, a strong antiviral drug^7^. The improved clinical outcome caused by the addition of ribavirin to antivirals has been also observed in several other viral infections, including HCV^8–11^, Junin and Pichinde virus^12^; Crimean Congo hemorraghic fever virus^13^, and Rift Valley fever virus^14^. However, the mechanisms by which ribavirin improves responsiveness when given in combination despite its low efficacy during mono-therapy remain poorly understood

Numerous modes of action (MOA) for ribavirin have been proposed (reviewed in ref.^15^): Ribavirin has been observed to act as an immunomodulatory agent by up-regulating specific interferon-stimulated genes^11,16^, and strengthening the adaptive antiviral immune response^17^. Ribavirin might also block viral production as it results in the impairment of the cellular enzyme IMP dehydrogenase (IMPDH) resulting in GTP depletion, and the direct inhibition of the HCV nonstructural 5B (NS5B) RNA-dependent RNA polymerase^15,18^. In addition, ribavirin has been characterized as a mutagen for HCV and hepatitis E virus (HEV), driving the virus to its error catastrophe^16,19–23^ and thereby limiting its specific transmission. Although there has been evidence supporting each of these hypotheses, the preeminent mode of action is still unresolved and might vary dependent on the type of viral pathogen studied.

In this study, we investigate to which extent the proposed various non mutually-exclusive roles of ribavirin affect the infection of LASV in mice. To this end, we develop mathematical models describing viral and infection dynamics incorporating ribavirin’s potential MOAs, including impairment of viral production, inhibition of cell damage, modulation of immune responses, and limitation of virus infectivity. We evaluate the ability of these models to describe experimental data on viral load and cell damage marker kinetics of LASV infected mice which were treated with mono-therapy of ribavirin or favipiravir, or with a combination of both drugs using sub-optimal doses^7^. Our analysis suggests that ribavirin mainly protects infected cells from dying, while having no effect on viral production or transmission.

## Results

### LASV infection data

**Figure 1:**
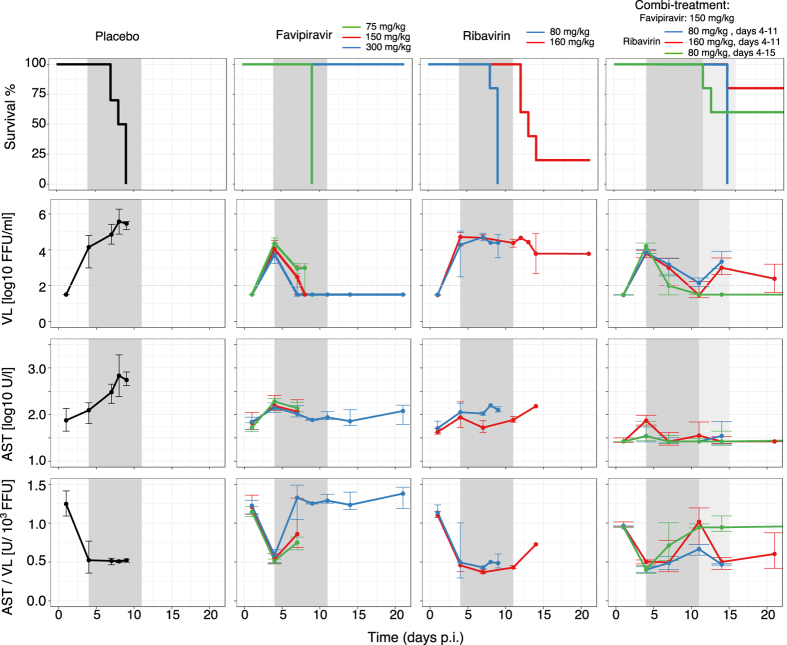
Survival (first row), median viremia (second row), median AST (third row), and AST:VL ratio (fourth row) in mice infected with 1000 FFU of Lassa Ba366. Animals were treated with PBS (left column), single doses of favipiravir or ribavirin (middle columns), and with three types of combination therapies (right column). Error bars represent the standard deviation. The dark and light gray areas depict the treatment period between 4–11, and 4–15 days post infection (p.i), respectively. Parts of this figure are adapted from ref.^7^.

While all placebo treated animals develop high viral titers and high levels of aspartate aminotransferase (AST), a marker for cell damage, there is a large variation in virus load (VL) and AST levels across different regimes of the mono- and combi-therapies (Fig. 1). Favipiravir reduces viremia in a dose-dependent manner, i.e. the higher the administered dose, the stronger the reduction in VL (Fig. 1, middle column). In contrast, ribavirin’s effect on viral titers is rather limited. Both drugs successfully limit the increase of AST, with ribavirin showing a larger decrease of AST relative to VL (Fig. 1, bottom row).

During combination-treatment, the time course of VL (Fig. 1, right column) is comparable to that observed in treatment regimes with 150 mg/kg favipiravir during mono-therapy, indicating that the effect on VL seems to be largely driven by favipiravir. Interestingly, the reduction in AST levels seems to be larger than that observed during mono-therapy (Fig. 1 third row). Taken together, the data suggest that ribavirin has little effect on viremia but seems to rather affect AST dynamics.

### Investigating ribavirin’s mode of action

Although there is large evidence for ribavirin in decreasing viral production *in vitro* ^24–27^, our data do not indicate a substantial effect of ribavirin on viral load during LASV infection *in vivo*, yet suggest that ribavirin clearly improves disease outcome during combination therapy. As the precise antiviral effect of ribavirin for LASV infection is unknown, we independently investigate four mechanisms of action using mathematical models describing viral and AST dynamics during LASV infection with and without treatment. The mathematical models follow the concentration of uninfected target cells, productively infected cells, and free infectious virions, as well as the levels of AST (for a detailed description of the model and the corresponding equations see *Materials and Methods*).

**Figure 2:**
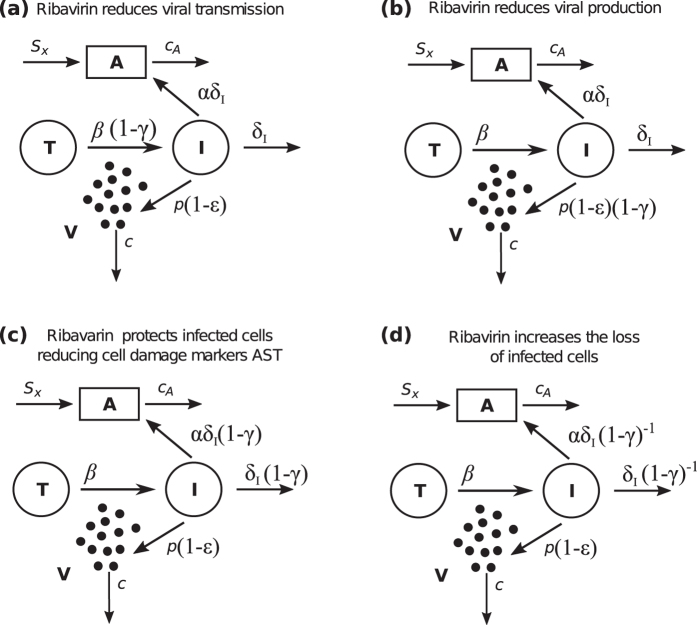
Schematic representation of possible drug effects on viremia and AST dynamics. The inhibitory activity of favipiravir and ribavirin are represented by *ε* and *γ*, respectively. Favipiravir inhibits viral production by decreasing the viral production rate *p*. We test several hypotheses regarding the unknown mode of action of ribavirin: (**a**) ribavirin affects the transmission of virions, *β* (model A), (**b**) ribavirin adds to the effect of favipiravir decreasing viral production, *p* (model B), (**c**) ribavirin decreases the death rate of infected cells (model C), and (**d**) ribavirin increases the death rate of infected cells *δ*_*I*_, e.g. by enhancing the host’s immune response (model D).

The different drug mechanisms are incorporated into the model by modifying the model parameters characterizing the hypothesized mode of action. As favipiravir is generally observed to inhibit the production of new infectious virions^28,29^, it is assumed to reduce the viral production rate *p* by a factor (1 − *ε*) (Fig. 2a). The four mutually exclusive mechanisms of ribavirin that are tested include (i) ribavirin’s activity as a mutagen, which by increasing the mutation rate reduces the infectivity of new virions and, thus, the transmission rate *β* (Fig. 2a); (ii) the reduction of the viral production rate *p* as ribavirin has been observed to inhibit HCV polymerase (Fig. 2b); (iii) the cell protective effect (i.e. reduction in AST) modeled by an inhibition of the death rate of infected cells (Fig. 2c); (iv) and the enhancement of antiviral immunity which is modeled by increasing the death rate of infected cells (Fig. 2d).

Fitting each of the different models to the data of LASV infected mice treated with mono-therapy of either ribavirin or favipiravir or left untreated, we find that the parameter estimates describing the general infection dynamics are similar in all models (Table 1). In all mice, the virus grows exponentially because of the large pool of available target cells (*T*_0_ = 10^6^ cells/ml) and their rapid infection. The basic reproduction number is estimated to *R*_0_ ≈ 5–6.0 (Table 1), with a viral production rate of *p* ≈ 2–4 FFU day^−1^ and an elimination rate of infected cells of *δ*_*I*_ ≈ 0.5 day^−1^, corresponding to a half-life of 1.3 days.

**Table 1:** Parameter estimates for viral dynamic models of favipiravir and ribavirin during LASV infection. *T*_0_ = 10^6^ cells/ml.

Parameters	Model A mean (r.s.e)	Model B mean (r.s.e)	Model C mean (r.s.e)	Model D mean (r.s.e)	Model C1 mean (r.s.e)	Model C2 mean (r.s.e)	Model C3 mean (r.s.e)
*V*_0_ (FFU/ml)	10.00	10.00	10.00	10.00	10.00	10.00	10.00
*T*_0_ (cells/ml)	10^6^	10^6^	10^6^	10^6^	10^6^	10^6^	10^6^
*c* (/day)	20.00	20.00	20.00	20.00	20.00	20.00	20.00
*c*_*A*_ (/day)	1	1	1	1	1	1	1
*R*_0_	6.72 (20)	6.08 (19)	4.96 (13)	5.86 (16)	5.62 (16)	4.92 (12)	4.62 (12)
*δ*_*I*_ (/day)	0.48 (23)	0.53 (22)	0.60 (16)	0.54 (19)	0.54 (19)	0.67 (15)	0.71 (14)
*p* (FFU/day)	3.52 (27)	3.9 (27)	1.82 (22)	4.06 (25)	3.09 (24)	3.19 (22)	2.34 (24)
*α* (×10^−4^)	14.1 (17)	12.3 (18)	18.0 (15)	11.2 (21)	22.0 (16)	23.0 (14)	19.3 (13)
*s*_*x*_ (U/l)	71.80 (6)	72.50 (6)	66.80 (6)	74.40 (6)	71.30 (6)	66.50 (6)	66.40 (6)
${{\rm{ED}}}_{{\rm{50}}}^{{\rm{F}}}$(mg/kg)	1.61 (50)	1.85 (52)	3.9 (49)	1.89 (46)	2.3 (47)	3.2 (46)	4.01 (47)
${{\rm{ED}}}_{{\rm{50}}}^{{\rm{R}}}$(mg/kg)	75.30 (36)	164.00 (31)	6.71 (37)	4.4×10^5^ (510)	9.05 (41)	4.93 (28)	6.95 (30)
${{\rm{ED}}}_{{\rm{50,2}}}^{{\rm{R}}}$ (mg/kg)					6.05 × 10^3^ (513)	73.80 (23)	72.8 (30)
*σ*_*a*_ (log_10_ FFU/ml)	0.61 (7)	0.62 (7)	0.71 (7)	0.59 (7)	0.65 (8)	0.64 (7)	0.67 (7)
*σ*_*b*_ (U/l)	0.31 (9)	0.31 (9)	0.22 (11)	0.32 (9)	0.27 (9)	0.21 (10)	0.21 (11)
BIC	2214.69	2219.70	2129.41	2227.8	2184.87	2146.22	2152.37

All parameters were estimated using non-linear mixed effect models. Here, *r.s.e* refers to the relative standard errors (%), *σ*_*a*_: standard deviation of additive residual error, and *σ*_*b*_: standard deviations of proportional residual error. For the meaning of the individual parameters see Fig. 2 and *Materials and Methods*.

The dynamics following the peak of the VL depend on the given treatment. Therefore, the major differences in parameter estimates are observed in parameters describing the effectiveness of the drugs, i.e., ${{\rm{ED}}}_{{\rm{50}}}^{{\rm{F}}}$, and ${{\rm{ED}}}_{{\rm{50}}}^{{\rm{R}}}$, which define the drug dose at which drug effectiveness is 50%. Favipiravir is estimated to be very effective in reducing the viral production rate, with ${{\rm{ED}}}_{{\rm{50}}}^{{\rm{F}}}$ values ranging between 1.6–4.0 mg/kg, indicating an effectiveness *ε* of 0.95–0.98 and 0.98–0.99, for doses of 75 mg/kg and 300 mg/kg, respectively. The efficacy of the drug is reflected by the exponential decline in VL observed after the peak. On the other hand, the efficacy of ribavirin varies depending on the mechanism of action studied (Table 1). Our analysis indicates that ribavirin is likely to be a potent agent in preventing infected cells from dying (model C, ${{\rm{ED}}}_{{\rm{50}}}^{{\rm{R}}}$ = 6.71 mg/kg), but is rather inefficient in reducing viral transmission (model A, ${{\rm{ED}}}_{{\rm{50}}}^{{\rm{R}}}$ = 77.90 mg/kg), or impeding viral production (model B, ${{\rm{ED}}}_{{\rm{50}}}^{{\rm{R}}}$ = 164.00 mg/kg). In addition, our analysis suggests that ribavirin does not lead to increased cell death due to enhanced immune responses (model D, ${{\rm{ED}}}_{{\rm{50}}}^{{\rm{R}}}$ = 6.05 × 10^3^ mg/kg).

**Figure 3:**
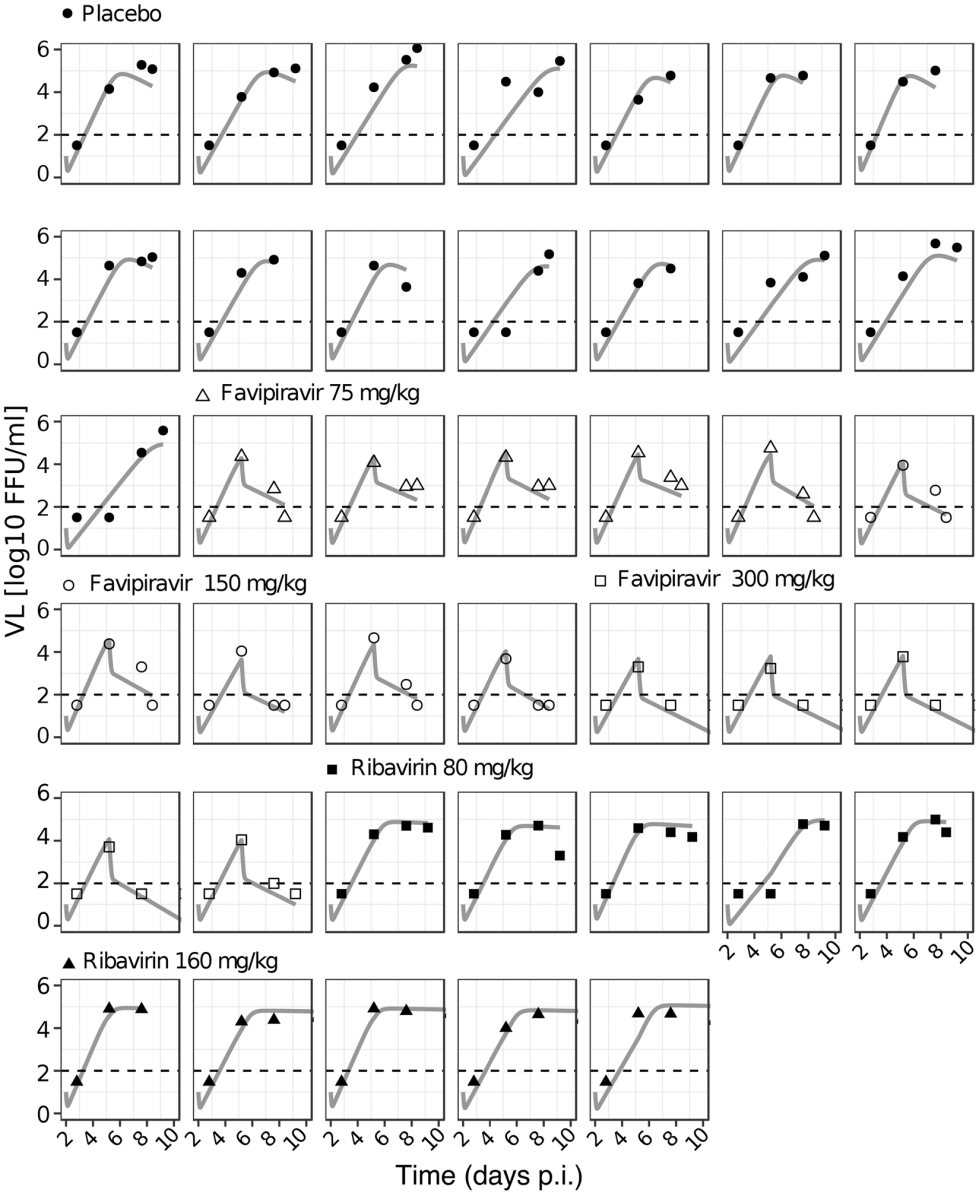
Individual data and model fits for VL dynamics until the end of treatment. Measured data are depicted in symbols, and the gray solid line shows the best fit of model C for each individual mouse. Depicted are all doses used for mono-therapy: PBS (placebo, filled circles); Favipiravir 75 (open triangles), 150 (open circles), and 300 (open squares) mg/kg; and ribavirin 80 (filled squares), and 160 (filled triangles) mg/kg.

**Figure 4:**
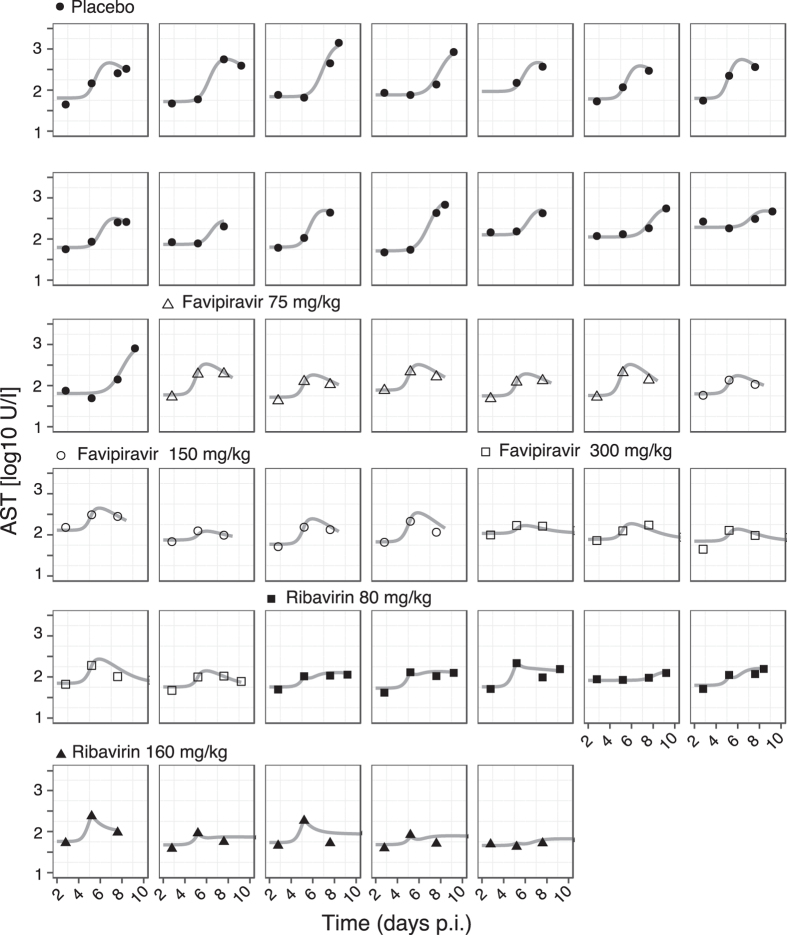
Individual data and model fits for AST dynamics. Measurements of AST levels are depicted in bullets, and the gray solid line shows the best fit of model C for each individual mouse. Symbol legend as in Fig. 3.

To determine the most likely mechanisms by which ribavirin affects viral dynamics in LASV infection, we rank these models according to their ability to describe the observed data by using the Bayesian Information Criterium (BIC), a measure used for model selection where the model with the lowest BIC is preferred. Among all the models tested, model C in which ribavirin protects infected cells from dying, thereby reducing the cell damage marker AST (Fig. 2c), describes the observed data best. Indeed, this model shows very good agreement between individual model predictions and observed data on VL and AST dynamics (Figs 3 and 4). Particularly, this model captures the “plateau” observed in the VL when mice are treated with ribavirin, which can be explained by the resulting longer life-span of infected cells, allowing for more viral production per cell. Note that the selection for model C is independent of the assumed initial condition for *T*_0_ as shown by univariate sensitivity analysis (Table 2).

**Table 2:** Sensitivity analysis for model comparisons.

	*T*_0_ = 10^6^(cells/ml)	*T*_0_ = 10^7^(cells/ml)
model A	2214.69	2204.92
model B	2219.70	2217.80
model C	2129.41	2126.40
model D	2227.8	2231.23

This sensitivity analysis was carried out for *T*_0_ with a fixed *V*_0_ = 10 FFU/ml. The BIC-values for the different models are shown.

Naturally, ribavirin might not be acting on a single mechanism alone, but could affect several aspects of the virus replication simultaneously. Therefore, having identified cell protection as ribavirin’s main MOA, we extend model C by adding additional modes of action (see *Materials and Methods*): (i) We investigate whether ribavirin might affect AST levels with a different efficacy as the life span of infected cells, and we test whether ribavirin, in addition to protecting infected cells from dying, might (ii) inhibit viral production or (iii) viral transmission. However, none of these model extensions (model C1-C3) showed an improved explanation of the observed dynamics compared to model C (see Table 1). In summary, our analysis suggests that ribavirin’s main MOA is to protect infected cells from dying, thereby reducing the release of cell damage markers in the circulation, rather than impairing viral transmission, viral production, or enhancing the host’s immune response.

### Predicting combination therapy

**Figure 5:**
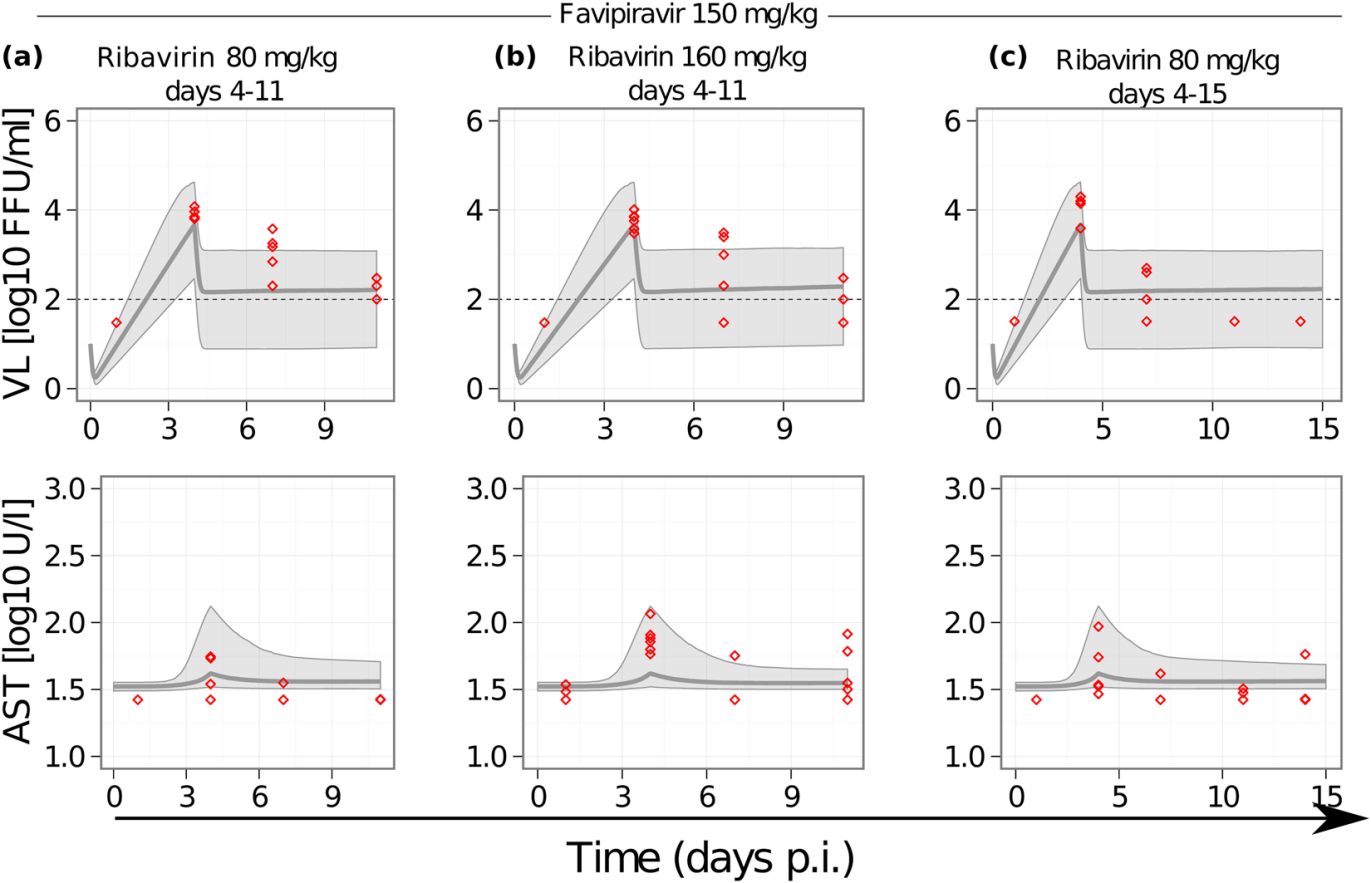
Model predictions for combination therapy. We simulate a short treatment (7 days) of LASV infected mice with combinations of 150 mg/kg favipiravir and 80 mg/kg ribavirin (**a**), 150 mg/kg favipiravir and 80 mg/kg ribavirin (**b**); and a long treatment period (11 days) with a combination of 150 mg/kg favipiravir and 80 mg/kg ribavirin (**c**). All treatment started at day 4 p.i.. Solid gray lines depict the median prediction out of 1000 simulations for VL (upper row) and AST (lower row) based on parameter ranges obtained for model C (Table 1). Shaded areas represent the 90% predictive intervals. Red circles represent the experimental data for the corresponding treatment regime as measured in ref.^7^.

To validate the proposed mechanism of action for ribavirin against LASV infection, we use the parameter estimates obtained for model C to predict the VL and AST dynamics during combination therapy with both drugs (Fig. 5a–c). For each combination therapy, we perform 1000 simulations sampling from the estimated parameter distributions and using the predicted efficacy for the corresponding drug doses. For all three combination-treatments used by Oestereich *et al*.^7^, our median predictions are in good agreement with the data for both, VL and AST dynamics alike (Fig. 5a–c). This observation supports the assumed inhibiting effect of ribavirin on reducing AST levels by protecting infected cells from dying as its potential mechanism of action.

## Discussion

Since its discovery in the early 70 s, ribavirin has been used for the treatment of several viral infections, including HCV, LASV, and respiratory syncytial virus (RSV)^30–35^. Despite its observed antiviral effect against LASV and HCV *in vitro*, the drug has no substantial effect on reducing viremia *in vivo*, but significantly improves clinical outcome when used in combination with other drugs. Many mechanisms of action have been proposed so far, especially regarding ribavirin’s role as a mutagen^19,20^, or as an immuno-modulatory agent^17^. These hypotheses rely on studies predominantly carried out in the context of HCV infection. But given ribavirin’s broad antiviral activity, it is important to study whether its effects might vary for other types of pathogens. Moreover, because of its clinical importance, it is crucial to determine its mechanisms of action, providing understanding of the differences in its effects during mono- and combination-therapy.

There is a large body of evidence suggesting that ribavirin is an important antiviral in the context of hemorrhagic fever viruses^12,13,34^, and it has been used as a standard therapy during LASV infection in humans^35^. Here, we investigate ribavirin’s effects on LASV *in vivo* by combining experimental data and mathematical modeling. This approach allows us to systematically test several mutually exclusive hypotheses for the mode of action of ribavirin. Our analysis suggests that ribavirin’s main mode of action against LASV infection is to protect infected cells from dying, providing a good explanation as to how ribavirin can decrease AST levels while hardly reducing virus titers. This is in agreement with experimental data, showing that mice treated with combined treatment experience less liver tissue damage compared to those treated with single doses of favipiravir^7^, indicating a cell-protective effect. Similar observations have been made during influenza virus infection, where ribavirin improved survival rate and lung pathology without reducing viremia in the lungs^36^. The potential mechanisms leading to the observed cell protective effect remain unresolved, but possible explanations include ribavirin’s modulation of immunological and pathophysiological pathways^37,38^, thereby inhibiting macrophage activation and cytokine production^37^; and the anti-proliferative effect on lymphocytes observed at high concentrations of ribavirin^39^. The discrepancy between ribavirin’s antiviral efficacy observed *in vitro* ^7^ and its main mechanism of action *in vivo* could be due to different factors, including the type of cells infected and the actual pharmacokinetics. In addition, inhibition of viral replication might still play a role *in vivo* (e.g. Model C2), although to a lesser extent.

Using the identified and quantified mode of action of ribavirin, our model predictions agree with the overall VL and AST dynamics observed in mice treated with combinations of both drugs (Fig. 5). Furthermore, while assuming independent effects, i.e., Bliss-independence of both drugs, our analysis supports previous hypotheses on the synergistic effect of ribavirin and favipiravir when given as a combination-therapy^7^. In their study, Oestereich *et al*. suggested that the combination of both drugs improves clinical outcome by direct suppression of viral replication (favipiravir) and modulation of immunological and pathophysiological pathways (ribavirin) as it has been observed previously^37,38^. Additionally, as favipiravir competes with GTP and ATP during RNA synthesis^28^, the depletion of the intracellular GTP pool by ribavirin^40^ may promote the incorporation of favipiravir into the viral RNA. The effect of a ribavirin-dependent activation of favipiravir needs to be tested and could possibly describe the observed downslope in VL after day 7 and the eradication of the virus, features that our current model cannot reproduce.

Our mathematical model describes VL dynamics in LASV infection during treatment, reproducing the strong antiviral effect of favipiravir reflected in the rapid decline in VL, as well as the high and stable viremia in mice receiving ribavirin. To apply this model to the experimental data, several simplifying assumptions were made. We base our calculation of the initial target cell population on liver-resident Kupffer cells as one of the main target cells, assuming a total size of *T*_0_ = 10^6^ cells/ml. Some studies suggest liver-resident macrophages in mice to be slightly more frequent, i.e., in the order of 5 × 10^7^ cells^41^. In addition, macrophages and monocytes are found in all tissues, explaining the wide distribution of LASV with viral replication observed in several organs including brain, lung, and heart tissue^7,42,43^. Thus, the target cell population might be actually larger than assumed, e.g., in the order of *T*_0_ = 10^7^ cells/ml. However, while *T*_0_ = 10^6^ cells/ml might underestimate the real value of target cells, it is consistent with our data. An increase would strongly affect the estimates of the viral production rate *p* leading to low production rates that would not be coherent with the estimated basic reproductive number *R*_0_ and the obtained half-life of infected cells (Table 3). In the context of the actual target cell pool size, estimates of parameters characterizing viral kinetics should be taken with care, such as the viral production rate *p*, which can only be estimated in combination with the target cell pool size *T*_0_ (see *Materials and Methods*), as well as the death rate of infected cells *δ*_*I*_. These parameters might also vary depending on the initial inoculum size *V*_0_ (Table 3), which was fixed to ensure parameter identifiability. Accordingly, identification and quantification of the actual target cell pool for LASV replication is essential for a proper quantification of LASV production rates. Nevertheless, our conclusion on ribavirin’s main mode of action remains robust when performing a sensitivity analysis on the target cell pool size *T*_0_ indicating that the model assuming that ribavirin acts by protecting infected cells from dying (Model C) consistently fits the data best (Table 2). In addition, our conclusions on parameter estimates were robust to the choice of the Hill coefficients for favipiravir and ribavirin measuring the level of drug molecule cooperation (Table 4).

**Table 3:** Sensitivity analysis for different values of the initial inoculum *V*_0_ and the initial target cell pool *T*_0_ for model C.

	*V*_0_ = 0.01(FFU/ml)	*V*_0_ = 0.10 (FFU/ml)	*V*_0_ = 1(FFU/ml)	*T*_0_ = 10^5^(cells/ml)	*T*_0_ = 10^7^(cells/ml)	*T*_0_ = 10^8^(cells/ml)
*R*_0_	15 (22)	11.7 (22)	7.07 (16)	6 (9)	5.34 (14)	5.11(13)
*δ*_*I*_(/day)	0.36 (21)	0.38 (22)	0.53 (18)	0.5 (9)	0.6 (16)	0.58 (16)
*p* (FFU/day)	1.42 (21)	1.45 (22)	1.74 (23)	16.5 (19)	0.18 (22)	0.02 (22)
*α* (×10^−4^)	11.8 (17)	13 (16)	14.1 (14)	140 (15)	1.9 (15)	0.19 (15)
*s*_*x*_ (U/l)	68.3 (6)	67.4 (6)	66.8 (6)	67.1 (6)	66.3 (6)	67.2 (6)
${{\rm{ED}}}_{{\rm{50}}}^{{\rm{F}}}$ (mg/kg)	1.65 (43)	1.46 (47)	2.19 (50)	2.34 (35)	2.96 (49)	3.41 (49)
${{\rm{ED}}}_{{\rm{50}}}^{{\rm{R}}}$ (mg/kg)	18 (46)	15.3 (42)	10.1 (35)	13.2 (37)	5.98 (34)	6.06 (39)
*σ*_*a*_ (log_10_ FFU/ml)	0.543 (9)	0.633 (9)	0.663 (8)	0.67 (7)	0.71 (7)	0.69 (8)
*σ*_*b*_ (U/l)	0.274 (11)	0.257 (11)	0.223 (11)	0.22 (11)	0.22 (11)	0.22 (11)
BIC	2169.67	2175.89	2173.73	2148.30	2126.40	2129.31

The mean of the estimated parameters with their respective relative standard errors﻿ (in %)﻿ are shown. For the analysis of *V*_0_, we fixed *T*_0_ to 10^6 ^cells/ml. Similarly, the sensitivity analysis for *T*_0_ was carried out with a fixed *V*_0_ = 10 FFU/ml.

**Table 4:** Sensitivity analysis for different values of Hill coefficients n_R_ for ribavirin in model C.

	n_F_ = 1			n_F_ = 3.5			
	n_R_ = 2	n_R_ = 3	n_R_ = 4	n_R_ = 1	n_R_ = 2	n_R_ = 3	n_R_ = 4
*R*_0_	5.13 (12)	4.66 (11)	5.29 (12)	4.77 (12)	5.29 (10)	4.88 (9)	5.72 (13)
*δ*_*I*_ (/day)	0.58 (14)	0.65 (14)	0.56 (13)	0.63 (14)	0.56 (15)	0.62 (10)	0.52 (14)
*p* (FFU/day)	1.88 (20)	2.02 (19)	1.89 (17)	1.86 (21)	1.84 (17)	2.04 (18)	1.84 (18)
*α* (×10^−4^)	18.2 (15)	17.5 (14)	18.5 (15)	18.2 (14)	1.81 (14)	16.5 (15)	17.2 (15)
*s*_*x*_ (U/l)	67.1 (6)	67.2 (6)	68.1 (6)	66.9 (6)	66.6 (6)	67.4 (6)	67.9 (6)
${{\rm{ED}}}_{{\rm{50}}}^{{\rm{F}}}$ (mg/kg)	3.46 (49)	4.88 (47)	3.43 (46)	5.61 (30)	38.6 (13)	43.3 (9)	39.2 (14)
${{\rm{ED}}}_{{\rm{50}}}^{{\rm{R}}}$ (mg/kg)	26.2 (15)	40.9 (11)	51.4 (11)	44.9 (14)	26 (15)	43.3 (13)	54 (11)
*σ*_*a*_ (log_10_ FFU/ml)	0.698 (7)	0.683 (8)	0.679 (7)	0.74 (7)	0.731 (7)	0.71 (7)	0.678 (7)
*σ*_*b*_ (U/l)	0.219 (11)	0.221 (11)	0.238 (11)	0.22 (11)	0.226 (11)	0.22 (11)	0.238 (11)
BIC	2193.73	2133.3	2146.5	2135.41	2136.98	2137.46	2145.93

For favipiravir, a Hill coefficient of n_F_ = 1 and n_F_ = 3.5^44^ was chosen.

The mean of the estimated parameters with their respective relative standard errors (in %)﻿ are shown. For the analysis, we expressed the dose-dependent- efficacies as: $\varepsilon ={{\rm{D}}}_{{\rm{F}}}^{{{\rm{n}}}_{{\rm{F}}}}/({{\rm{D}}}_{{\rm{F}}}^{{{\rm{n}}}_{{\rm{F}}}}{+(\mathrm{ED}}_{{\rm{50}}}^{{\rm{F}}}{)}^{{{\rm{n}}}_{{\rm{F}}}})$ for favipiravir, and $\gamma ={{\rm{D}}}_{{\rm{R}}}^{{{\rm{n}}}_{{\rm{R}}}}/({{\rm{D}}}_{{\rm{R}}}^{{{\rm{n}}}_{{\rm{R}}}}+{{(\mathrm{ED}}_{{\rm{50}}}^{{\rm{R}}})}^{{{\rm{n}}}_{{\rm{R}}}})$ for ribavirin. This sensitivity analysis was carried out with fixed *T*_0_ = 10^6^ cells/ml and *V*_0_ = 10 FFU/ml.

Although LASV rapidly results in a severe and often fatal hemorrhagic fever, the estimates of viral kinetic parameters differ to those recently estimated during Ebola infection^44^. Particularly, our estimates for the basic reproduction number *R*_0_ (estimated to be ~6) remained lower than the reproductive number found in mice infected with EBOV (estimated to be ~9). Similarly, the half-life of infected cells in our model is estimated to be 1.3 days, much larger than the 6.4 h estimated for Ebola^44^. Note, that these parameter estimates might be affected by the initial inoculum size *V*_0_, as well as the time resolution of the measured viral kinetics.

As there is yet no clear characterization of the immune response during LASV infection^45^, we can only implicitly include it in the clearance rate of the virus *c*, and the loss rate of infected cells, *δ*_*I*_. Only when the dynamics of immune responses involved in the clearance of LASV are elucidated, more detailed models can be developed to explicitly analyze ribavirin’s potential effect on the modulation of the immune response^37,38^.

In combination with favipiravir, ribavirin has a clear beneficial effect against LASV infection *in vivo*, reflected in the increased survival of those animals treated with a combination of both drugs (Fig. 1). However, given that the levels of viremia and AST are similar to those observed during the corresponding mono-therapies (during which the survival rate was close to zero), the improved disease outcome provided by the additional treatment with ribavirin seems surprising. This indicates that viral load and AST dynamics might not be sufficient predictors for clinical outcome during LASV infection. Additional mechanisms might be pivotal during the course of infection, including for example vascular leakage, the time courses of the different compartments targeted by the virus, as well as the involved immune responses. It is possible that ribavirin is also acting on any of these mechanisms thereby leading to a better and faster recovery. Understanding the determinants for the improved disease progression is therefore crucial to fully understand ribavirin’s mode of action and its contribution to survival.

Finally, ribavirin’s mechanism of action indicated here for LASV infection might be representative for infections caused by other hemorrhagic fever viruses. The beneficial effect of ribavirin in combination with favipiravir has already been elucidated during infections with, among others, Crimean Congo hemorrhagic fever virus and Junin virus^12^. Given that favipiravir also shows strong antiviral effects against EBOV^44^, it remains to be determined whether the addition of ribavirin to the treatment with favipiravir would improve the outcome of current therapeutic regimes against EBOV infection.

## Methods

### LASV infection data

The experimental data have been described previously in ref.^7^. In brief, chimeric *Ifnar* ^−/−*B*6^ C57BL/6 mice were infected intraperitoneally (i.p.) with 1000 focus-forming units (FFU) of LASV Ba366. Signs of disease, body weight, and body temperature, as well as levels of aspartate aminotransferase (AST) and alanine aminotransferase (ALT), and infectious virus particles were measured in the blood every 3–7 days over a period of 21 days^7^. Viral load was quantified in immunofocus assay as FFU, with a limit of detection of 2 log_10_ FFU/ml.

Mice were separated into groups treated with different doses of ribavirin and/or favipiravir. Doses used comprise treatment with 80 mg/kg (*n* = 5) or 160 mg/kg (*n* = 5) of ribavirin administered daily by the i.p. route; favipiravir at 75 (*n* = 5), 150 (*n* = 5), or 300 (*n* = 5) mg/kg administered twice daily per os using a stomach probe; and for control, placebo-treated mice (*n* = 12) received twice daily PBS (Fig. 1). All treatments started at day 4 p.i. and continued for 7 days or until death.

Two different combination therapies were tested using 150 mg/kg favipiravir and 80 mg/kg ribavirin (combi-therapy 1), or 150 mg/kg favipiravir and 160 mg/kg ribavirin (combi-therapy 2), with the same administration routes for each drug as during mono-therapy. Similar to the mono-therapy treatments, the combination of drugs was administered between days 4–11 p.i. Additionally, the effect of a longer treatment of combi-therapy 1 (i.e., administered until day 15 p.i.) was tested.

All animals treated with 75 and 150 mg/kg favipiravir or 80 mg/kg ribavirin, or left untreated died within 10 days p.i. In contrast, mice treated with 300 mg/kg of favipiravir or 160 mg/kg ribavirin, or with combination treatment had a higher chance of survival (Fig. 1).

All experiments were carried out in strict accordance with the recommendations of the German Society for Laboratory Animal Science under supervision of a veterinarian. The protocol was approved by the Committee on the Ethics of Animal Experiments of the City of Hamburg (permit no. 125/12). For a detailed statement on the procedures see *Supporting Information* in Oestereich *et al*.^7^.

### Mathematical model for virus and AST dynamics

To determine the mechanisms of action of ribavirin against LASV infection and to quantify its effect, we developed a mathematical model describing viral and infection dynamics including various hypotheses regarding potential drug effects. The within-host infection dynamics during LASV infection are described by the standard model of viral dynamics extended with the dynamics of AST levels as given in the following equations:

1 $$\frac{dT}{dt}=-\beta VT$$

2 $$\frac{dI}{dt}=\beta VT-{\delta }_{I}I$$

3 $$\frac{dV}{dt}=pI-cV$$

4 $$\frac{dA}{dt}={s}_{x}+\alpha {\delta }_{I}I-{c}_{A}A\mathrm{.}$$

Here, *T*, *I* and *V* denote the concentrations of uninfected target cells, productively infected cells, and infectious free virions, respectively. Free virions infect target cells at a rate *β* and are cleared at a rate *c*. Productively infected cells produce new infectious virions at a rate *p* and have an average half-life of *ln*(2)/*δ*_*I*_. Furthermore, we assume that the level of AST, denoted by *A*, is described by a constant source *s*_*x*_ and a clearance rate *c*_*A*_. In addition, AST is released from dying infected cells with a factor *α*.

#### Including treatment effects

With the exception of the placebo group, the model described in Eqs (1)–(4) needs to be extended in order to include the effect of the drug treatment started at day 4 p.i.

Based on previous observations, we assume that treatment with favipiravir reduces the viral production rate *p* by a factor (1 − *ε*), where *ε* describes the dose-dependent effectiveness of the drug, with *ε* = 1 being a drug that completely blocks viral production. The effectiveness *ε* of the treatment is modeled with an E_max_-model in a dose-dependent manner, i.e., $\varepsilon ={{\rm{D}}}_{{\rm{F}}}{/(D}_{{\rm{F}}}+{{\rm{ED}}}_{{\rm{50}}}^{{\rm{F}}})$, where D_F_ is the given dose of favipiravir and ${{\rm{ED}}}_{{\rm{50}}}^{{\rm{F}}}$ the dose of favipiravir at which 50% of the viral production has been blocked.

As the exact mechanism of action of ribavirin is unknown, we test different hypotheses by incorporating them within our model (see Fig. 2). Analogous to favipiravir, we assume that the effect of ribavirin occurs in a dose-dependent manner with $\gamma ={{\rm{D}}}_{{\rm{R}}}{/(D}_{{\rm{R}}}+{{\rm{ED}}}_{{\rm{50}}}^{{\rm{R}}})$ describing the dose-dependent effectiveness, with D_R_ the dose given and ${{\rm{ED}}}_{{\rm{50}}}^{{\rm{R}}}$ the dose at which the effectiveness is 50%.

**Model A: Limiting viral transmission:** One way of ribavirin to inhibit viral dynamics and disease progression is by inhibiting the viral transmission rate *β*. We incorporate this hypothesis into our model by replacing *β* in the equations by $\tilde{\beta }=\mathrm{(1}-\gamma )\beta$.

**Model B: Limiting viral production:** Ribavirin has been shown to inhibit the HCV polymerase, and to reduce GTP pools within the cell, affecting the capping efficiency of some RNA viruses ^46,47^. Therefore, similar to the assumption for favipiravir, we assume that ribavirin is also affecting the viral production rate. In this case, the viral production rate *p* in Eq. (3) is replaced by $\tilde{p}=p\mathrm{(1}-\varepsilon \mathrm{)(1}-\gamma )$, where *ε* describes the effectiveness of favipiravir while *γ* determines the effectiveness of ribavirin. Thus, we assume Bliss independence (i.e. both drugs have independent mechanisms of action) to describe the combined effectiveness.

**Model C: Modulating cell damage:** To incorporate the hypothesis that ribavirin has a cell protective effect, we assume that it prevents infected cells from dying. To this end, the death rate of infected cells *δ*_*I*_ is replaced by ${\tilde{\delta }}_{I}={\delta }_{I}\mathrm{(1}-\gamma )$, where *γ* denotes the dose dependent effectiveness of ribavirin in reducing infected cell death. A reduction in *δ*_*I*_ would also lead to a decreased production of AST.

We also test different extensions of model C. In model C1, we assume two separate dose-dependent effects of ribavirin on the death rate of the infected cells (*γ*_1_) and the increase in the AST levels (*γ*_2_). In this case, Eqs (2) and (4) change to

5 $$\frac{dI}{dt}=\beta VT-{\delta }_{I}\mathrm{(1}-{\gamma }_{1})I$$

6 $$\frac{dA}{dt}={s}_{x}+\alpha {\delta }_{I}\mathrm{(1}-{\gamma }_{2})I-{c}_{A}A\mathrm{.}$$

In a second extension, we additionally allow ribavirin to affect viral production, i.e., extending model C by additionally affecting the viral production rate by a factor (1 − *γ*_2_) (model C2). A third extension (model C3), combines model C and model A, i.e., additionally assuming that ribavirin also affects viral transmission.

**Model D: Increasing infected cell loss:** In contrast to model C, we incorporate here the hypothesis that ribavirin might be increasing the death rate of infected cells due to an increased immune response. This is incorporated into the model by replacing *δ*_*I*_ within Eqs (1)–(4) by ${\tilde{\delta }}_{I}={\delta }_{I}\mathrm{/(1}-\gamma )$, with *γ* ∈ [0, 1) denoting the dose dependent effectiveness of ribavirin.

### Parameter estimation based on mono-therapy data

We fitted each of the different models to the data of all LASV infected mice being treated with mono-therapy of either ribavirin or favipiravir or left untreated. Because they cannot be identified, the clearance rates of free virus, *c*, and AST, *c*_*A*_, are fixed without loss of generality to 20 day^−1^ ^44^ and 1 day^−1^, respectively. Because the parameters *p*, *β*, and *T*_0_ (the initial pool of target cells) are strongly correlated, we parameterize the infectivity as $\beta =\tfrac{{R}_{0}c{\delta }_{I}}{p{T}_{0}}$. With this parameterization, we are able to directly estimate the basic reproductive number *R*_0_, which is defined as the number of infected cells generated by one infected cell during its lifetime at the start of infection, i.e., before any depletion of target cells. Note that because of the high correlation of the before mentioned parameters, only the product *pT*_0_ can be estimated. Therefore, the estimated rate of viral production is directly proportional to the value assumed for *T*_0_.

The liver is assumed to be a main site of replication for LASV infection^2,3,7^, with tissue-resident macrophages, i.e. Kupffer cells, representing the major population of target cells^48^. The total number of Kupffer cells in a mouse can be estimated to be in the order of ~5 × 10^6^–5 × 10^7^ cells^41,49^. Assuming that LASV can distribute throughout the ~4 ml of extracellular fluid and that the body weight of an average mouse is approximately 20 g, we normalize the population of liver-resident macrophages as has been done previously^50^ leading to ${T}_{0} \sim {10}^{6}\mbox{--}{10}^{7}$ cells/ml. In the following, we set our initial target cell population to *T*_0_ = 10^6^ cells/ml. The sensitivity of our results is checked with regard to this choice.

In a first attempt to estimate the viral dynamics during LASV infection, we fit the model described in (1)–(4) to the data of placebo treated animals. A subsequent profile-likelihood analysis revealed that the best fits were obtained for an initial infection dose of *V*_0_ = 10 FFU/ml. Therefore, the initial viral concentration *V*_0_ was fixed to this value. In order to assess the stability of the results with regard to these parameter choices, a sensitivity analysis was carried out for different values of *T*_0_ and *V*_0_ (Table 3). Unless stated otherwise, we fix four parameters (i.e., *T*_0_ = 10^6^ cells/ml, *V*_0_ = 10 FFU/ml, *c* = 20 day^−1^, and *c*_*A*_ = 1 day^−1^), estimating all other parameters determining infection and AST dynamics (i.e., *R*_0_, *δ*_*I*_, *p*, *α*, *s*_*x*_), as well as drug effects (i.e., ${{\rm{ED}}}_{{\rm{50}}}^{{\rm{F}}}$ and ${{\rm{ED}}}_{{\rm{50}}}^{{\rm{R}}}$).

All parameters are estimated using non-linear mixed effect models. Hereby, the observation *O*_*ij*_ representing the observed viral load or AST level of subject *i* at time *t*_*ij*_ is given by:

7 $${O}_{ij}=f({\theta }_{i},{t}_{ij})+{e}_{ij},$$

where *f* is the function describing the model, *θ*_*i*_ is the vector of parameters for subject *i* and *e*_*ij*_ defines the residual error. The individual parameters are assumed to follow a log-normal distribution, hence, *θ*_*i*_ = *μ*exp(*η*_*i*_) where *μ* describes the fixed effects, representing population values, and *η*_*i*_ denotes the individual random effect supposed to follow a normal distribution with *N*(0, *ω* ^2^), where *ω* is the standard deviation of the random effect. The residual error, *e*_*ij*_, follows a normal distribution with $N\mathrm{(0,}\,{\sigma }_{ij}^{2})$. Here, we assume an additive model for the log_10_ VL and a proportional error for AST.

The model parameters were estimated in MONOLIX 4.2.6. using the SAEM algorithm accounting for data below the limit of detection, i.e., for the VL at 2 log_10_FFU/ml. All fits were performed until the last day of treatment, i.e., day 11, or the time of death if death occurred before. Model comparison was performed according to the Bayesian Information Criterium (BIC), a measure used for model selection which is based on the log-likelihood function of the model and penalized by the number of observations and parameters (the model with the lowest BIC is preferred).

### Predictions to the combi-therapy

We use the parameter estimates obtained from the analysis of the mono-therapy data to predict the viral and AST dynamics during the different combi-treatments. For each combination therapy, we sample from the estimated parameter distributions obtained for the corresponding dosages for ribavirin and favipiravir during mono-therapy, and compute the median and the 90% predictive intervals over 1000 individual simulations. We predict VL and AST dynamics for the three different experimental scenarios^7^: Combi 1 (150 mg/kg favipiravir + 80 mg/kg ribavirin) administered between days 4–11 or 4–15, and Combi 2 (150 mg/kg favipiravir + 160 mg/kg ribavirin) administered between days 4–11.

We found a significant difference in the baseline AST levels (i.e. at day 0 post infection) among those animals treated with mono-therapy (median 56 U/l) and those given a combination of both drugs (median 26.5 U/l, *p* ≤ 0.0005). To allow for comparisons of model predictions (Fig. 5), we scale the data from the combination-therapy by 2.8, the factor between the baseline AST levels *observed* during combi-therapy (26.5 U/l), and those *estimated* during the fitting procedure to the mono-therapies, i.e., *s*_*x*_ ≈ 69 U/l (Table 1).

### Statistical analyses

Differences in baseline AST were analyzed using two-tailed Mann-Whitney U test in the ${\mathbb{R}}$-software of statistical computing^51^.
